# Correct anteroposterior patterning of the zebrafish neurectoderm in the absence of the early dorsal organizer

**DOI:** 10.1186/1471-213X-11-26

**Published:** 2011-05-16

**Authors:** Máté Varga, Shingo Maegawa, Eric S Weinberg

**Affiliations:** 1 Department of Biology, University of Pennsylvania, Philadelphia, PA 19104, USA; 2 Current Address: Department of Genetics, Eötvös Loránd University, Pázmány Péter sétány 1/C, Budapest H-1117, Hungary; 3 Current Address: Dept. of Intelligence Science and Technology, Graduate School of Informatics, Kyoto University, Yoshidia-Honmachi, Sakyo, Kyoto 606-8501, Japan

## Abstract

**Background:**

The embryonic organizer (i.e., Spemann organizer) has a pivotal role in the establishment of the dorsoventral (DV) axis through the coordination of BMP signaling. However, as impaired organizer function also results in anterior and posterior truncations, it is of interest to determine if proper anteroposterior (AP) pattern can be obtained even in the absence of early organizer signaling.

**Results:**

Using the ventralized, maternal effect *ichabod *(*ich*) mutant, and by inhibiting BMP signaling in *ich *embryos, we provide conclusive evidence that AP patterning is independent of the organizer in zebrafish, and is governed by TGFβ, FGF, and Wnt signals emanating from the germ-ring. The expression patterns of neurectodermal markers in embryos with impaired BMP signaling show that the directionality of such signals is oriented along the animal-vegetal axis, which is essentially concordant with the AP axis. In addition, we find that in embryos inhibited in both Wnt and BMP signaling, the AP pattern of such markers is unchanged from that of the normal untreated embryo. These embryos develop radially organized trunk and head tissues, with an outer neurectodermal layer containing diffusely positioned neuronal precursors. Such organization is reflective of the presumed eumetazoan ancestor and might provide clues for the evolution of centralization in the nervous system.

**Conclusions:**

Using a zebrafish mutant deficient in the induction of the embryonic organizer, we demonstrate that the AP patterning of the neuroectoderm during gastrulation is independent of DV patterning. Our results provide further support for Nieuwkoop's "two step model" of embryonic induction. We also show that the zebrafish embryo can form a radial diffuse neural sheath in the absence of both BMP signaling and the early organizer.

## Background

The body plan of developing animal embryos is initially generated by establishment of the anteroposterior (AP) and dorsoventral (DV) axes. The dorsal organizer (i.e., Spemann organizer and homologous structures) is clearly important in formation of the DV axis (reviewed in [1-3]), but its role in AP axis development has been controversial (reviewed in [4,5]). Failure to form the Spemann organizer in frogs and fish [6-8] results not only in the absence of dorsal tissues, but also in the loss of anterior regions of the embryo. Nevertheless, there is also evidence that at least some degree of proper AP patterning occurs with surgical removal or genetic ablation of the organizer in mouse, chick, and zebrafish embryos [9-17]. In zebrafish and *Xenopus *embryos unable to form a dorsal organizer, head neurectodermal markers are still expressed in proper relative AP order if BMP signaling is absent [8,18,19]. The orientation of the AP axis with respect to the animal/vegetal (AnVeg) axis has also been disputed. Some have argued for the equivalence of the AP axis with the "classic" DV axis of anamniotes [20-22], while others have proposed a concordance of the AP and AnVeg axes in these groups [5]. As the function of the organizer may obscure an underlying mechanism that establishes AP pattern, we chose to further study the control of AP axis formation in embryos genetically blocked in the ability to form a dorsal organizing center.

Embryos bred from females homozygous for the *ich *mutation (*ich *embryos) show a reduction of maternal *β-catenin-2 *expression. Furthermore, treatment of wild-type embryos with a morpholino antisense oligonucleotide (MO) targeting *β-catenin-2 *causes a failure of organizer formation and loss of anterior tissues, whereas loss of *β-catenin-1 *alone has no ventralizing or posteriorizing effects [8]. Using a TOP-GFP Wnt-reporter line, we showed that while the MO against *β-catenin-2 *(βcat2MO) could eliminate the dorsal marginal expression of the transgene in shield stage embryos, a MO against *β-catenin-1 *(βcat1MO) had no effect in this region [23]. In contrast, the germ-ring transgene expression was abolished only when the two MOs were administered together, showing that this domain of expression was mediated redundantly by both *β*-catenins [23]. Injection of both MOs into wild-type embryos, or of βcat1MO to *ich *embryos already deficient in *β-catenin-2 *expression, caused the ectopic induction of *chordin *(*chd*) and *noggin1 *(*nog1*) around the blastodermal margin of the embryo [8,23]. Such embryos deficient in both β-catenins develop a distinctive phenotype at 24 hpf (termed 'ciuffo'), in which a protrusion of tissue from the vegetal end of the yolk expresses neurectodermal markers in an apparently proper AP pattern. This expression is dependent on *chd *[8,23]. The massive expression of *chd *in βcat1MO + βcat2MO-treated embryos would be expected to result in a marked inhibition of BMP signaling due to the direct binding of Chd to BMP ligand [24].

In the work presented here, we first show that *ich *embryos injected with βcat1MO + βcat2MO or with bmp2bMO alone both exhibit loss of BMP signaling and upregulate *chd*. Previously, we demonstrated that in 'ciuffo' embryos, key specific markers of the early organizer are never induced [8] and expression of *goosecoid *(*gsc*) [25] and *chd *are dependent on the endogenous, germ-ring expression of Nodal homologues [23].

Here we provide definitive evidence that the upregulation of *chd *in bmp2bMO-treated *ich *embryos is not due to the ectopic induction of organizer tissue, since other typical markers of the early and late dorsal signaling center are not induced. The ectopic expression of *chordin *appears to be a consequence of downregulation of BMP signaling, and not due to radialization of an early dorsal organizing center. Using embryos at several developmental stages, we show that both anterior and posterior neurectodermal markers are expressed with correct AP pattern, even in the absence of the organizer, as long as BMP signaling is inhibited. We then show that the same pathways that are involved in setting up the AP neurectodermal pattern in wild-type embryos - Wnt-, Nodal-, and FGF signaling - are required for elaboration of the full AP pattern in the absence of BMP and organizer signals. Finally, we examined the morphology of the neurectodermal tissue in 'ciuffo' embryos and found that cells with neuronal identity are organized in a sheath of mesoderm and endoderm, similar to the neural net present in cnidarians. We speculate that during the evolution of bilaterian precursors, the establishment of a DV-oriented BMP signaling gradient during embryogenesis resulted in the transformation of an outer radially organized, AP-patterned neural sheath, into the stereotypical vertebrate neural tube. This view extends the recent analysis of Meinhardt [5], which regards the generation of AP pattern of the vertebrate brain as an organizer-independent, ancestral, radially symmetric system.

## Results

### Embryos deficient in canonical Wnt signaling show loss of BMP signaling

**Figure 1:**
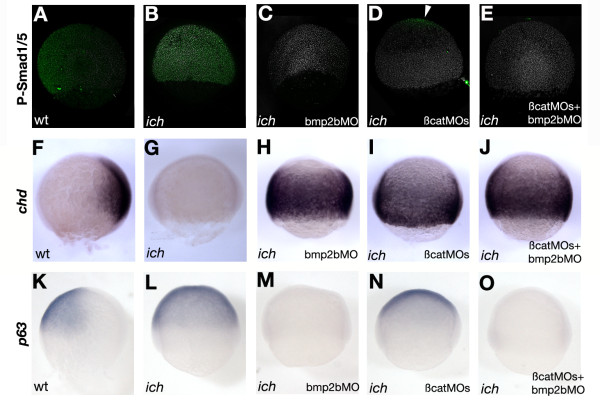
**Two independent ways of inhibiting BMP signaling result in massive ectopic expression of chordin**. (A-E) BMP signaling detected by immunohistochemistry using an anti phospho-Smad1/5/8 antibody; green dots indicate nuclei containing the phosphorylated Smad. (F-J) *In situ *hybridization with *chd *probe. (K-O) Expression of the epidermal marker *p63*. Wild-type embryos (A,F,K) are shown with in lateral view with ventral side to the left. Untreated *ich *embryos (B,G,L), and *ich *embryos injected with bmp2bMO (C,H,M), or with the two βcatMOs (D,I,N), or with all three MOs (E,I,O) are shown in lateral views. All embryos are at ~70% epiboly.

To better understand the effect of inhibition of Wnt/β-catenin signaling on formation and patterning of neurectoderm in the zebrafish embryo, we examined to what degree BMP signaling was affected by the elimination of expression of the two β-catenins by administration of βcat1MO + βcat2MO to *ich *embryos. As we had previously shown that such inhibition results in a high level of ectopic *chd *expression, we expected to find a very low level of BMP signaling in these embryos. We were also interested in whether the resulting inhibition of BMP signaling was equivalent to direct inhibition of BMP expression attained by injection of an MO against *bmp2b *(bmp2bMO) [26]. The degree of BMP signaling in these embryos was visualized by examining the distribution of phosphorylated Smad1/5 (P-Smad5), an indicator of cells actively transducing BMP signaling [27,28]. Administration of the two β-catenin MOs to *ich *embryos was in fact as effective as treatment of these embryos with bmp2bMO in eliminating BMP signaling in all regions except at the very animal pole (Figure 1). Wild-type embryos at 50% epiboly exhibit a gradient of nuclear P-Smad5 with the most intense staining at the ventral-most area of the embryo, and exclusion of P-Smad5 from the dorsal side (Figure 1A; [29]) where *chd *is expressed (Figure 1F). *ich *embryos show an expansion of P-Smad5 throughout the embryo, with no evidence of an activity gradient (Figure 1B) or of *chd *expression (Figure 1G). Injection of bmp2bMO into *ich *embryos at a concentration which phenocopies the *swirl *(*swr*) mutation in wild-type embryos [30,31], eliminates P-Smad5 in the embryo (Figure 1C), and results in a massive expression of *chd *(Figure 1H). We observe a similar phenotype when large amount of *chd *mRNA is injected into *ich *embryos (not shown). The loss of Bmp2b activity is known to impair expression of *bmp4 *and *bmp7 *[32,33]; thus, it is not unexpected that global BMP-signaling is lost in the bmp2bMO-treated embryos. Injection of *ich *embryos with βcat1MO + βcat2MO has very much the same effect on P-Smad5 (Figure 1D) and *chd *expression (Figure 1I) as bmp2bMO injection, except that nuclear P-Smad5 can be seen at the animal pole (Figure 1D arrowhead). Injection with the three MOs results in embryos with a distribution of P-Smad5 and *chd *expression very much as in embryos injected with bmp2bMO alone.

Ectopic expression of *chd *is also correlated with the repression of epidermal markers, as posited by the "neural default model" [34]. The ubiqitous expression of the epidermal marker *p63 *[35] observed in *ich *embryos (Figure 1L) is reduced upon the injection βcatMOs (Figure 1N), and completely abolished when bmp2bMO is used alone or in combination with the other MOs (Figure 1M,O). This latter result shows that in the absence of BMP signaling ectodermal cells can not acquire epidermal fates.

In summary, two different methods - injection of *ich *embryos with bmp2bMO or with βcat1MO + βcat2MO (i.e., 'ciuffo' embryos) - can be utilized to obtain embryos lacking the organizer and BMP signaling.

### Impairment of BMP-signaling does not result in ectopic organizer formation

**Figure 2:**
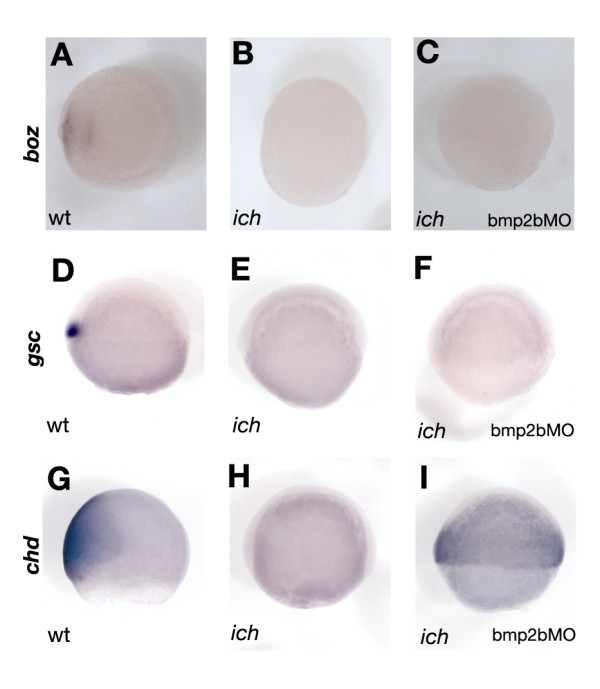
**Inhibition of BMP signaling in *ich *embryos does not result in organizer induction**. *In situ *hybridization with *boz *(A,B,C), *gsc *(D,E,F) and *chd *(G,H,I) probes in uninjected *ich *and bmp2bMO-injected *ich *embryos demonstrate that while impaired BMP signaling results in the transcriptional derepression of *chd *at the margins of the embryo (H,I), other characteristic organizer genes such as *boz *and *gsc *are not induced (C and F). (In A-C dome staged embryos are shown from an animal pole view, D-F and G-I present lateral views of 50% epiboly and 70% epiboly staged embryos, respectively. For wild-type embryos, dorsal is on the left.)

One important question to address is whether the induction of *chd *in MO-treated *ich *embryos reflects the ectopic induction of organizer tissue, or it is a transcriptional consequence of global BMP signaling downregulation. Our previous results showed that in 'ciuffo' embryos one of the earliest markers of endogenous organizer induction, *bozozok*/*dharma *(*boz*) [36-38] is never expressed, and the circumferential, germ-ring expression of later dorsal markers such as *gsc *and *chd *is induced with a significant delay (at 50% epiboly instead of at 30% epiboly) compared to their dorsal appearance in wild type embryos [8,23]. We now examined the expression of these markers in bmp2bMO-injected embryos (Figure 2). In contrast to *chd *(Figure 2I), expression of *boz *(Figure 2C) and *gsc *(Figure 2F) was not detected in such embryos.

Unlike some other components of the early DV transcriptional network (e.g. *squint *(*sqt*) [15] and *fgf8 *[39]) that are first expressed dorsally at sphere stage and then followed by a pan-germ-ring upregulation at ~30% epiboly, *boz *and *gsc *are exclusively expressed in the dorsal side of developing wild type embryos, and are thus more definitive markers of the early dorsal signaling center and organizer. Furthermore, while the ectopic expression of these genes can induce a complete axis in zebrafish [40,41], the overexpression of *chd *alone cannot do so, neither in wild type [41], nor in *ich *embryos (our unpublished observations). As is the case also in *Xenopus *[42], coinjection of *chd *mRNA with a Wnt-antagonist can induce anterior neural structures and notochord (our unpublished observations). But, unlike *Xenopus*, where *chd *is found to be necessary for the induction of a complete secondary axis [43], in zebrafish *chd *is dispensable for *gsc-*induced secondary axis formation [41]. These results offer clear evidence that the ectopic expression of *chd *in bmp2bMO-injected *ich *embryos is due to a global de-repression of its transcription in the absence of BMP signaling [44], and is not the consequence of ectopic induction of organizer tissue.

### Inhibition of BMP signaling reveals normal AP neurectodermal patterning in the absence of the organizer

**Figure 3:**
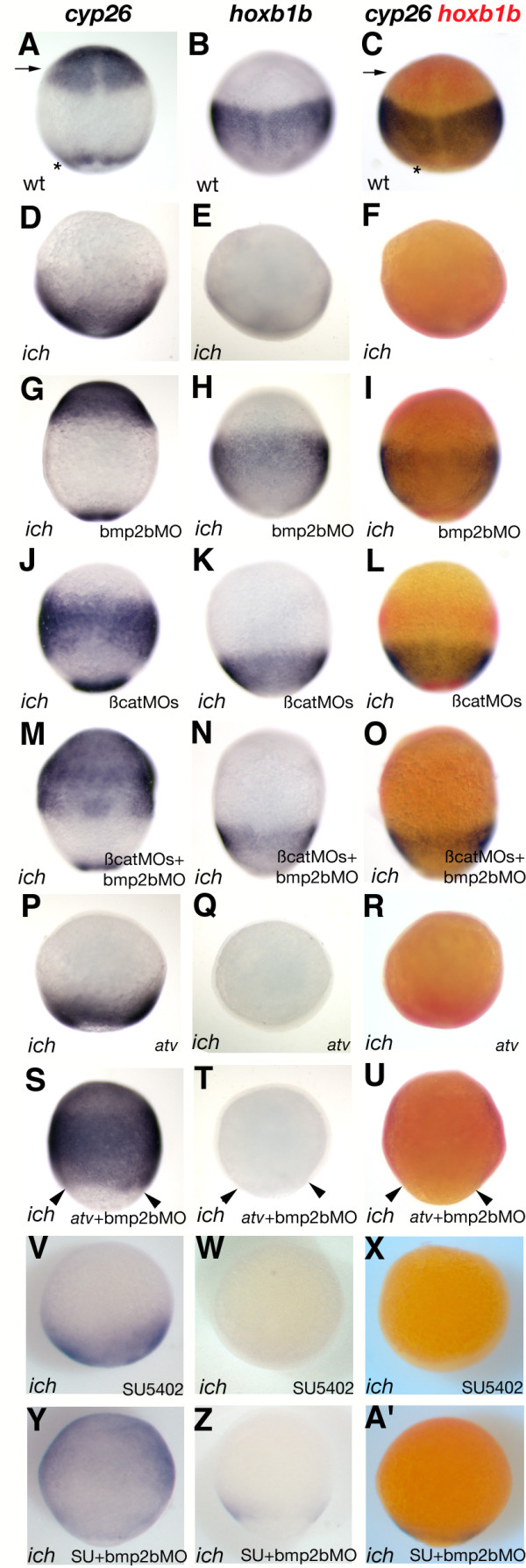
**Embryos inhibited in both BMP signaling and organizer function form neuroectoderm with correct AP pattern at the end of gastrulation and this patterning can be modulated by similar mechanisms as in wild-type embryos**. Single *in situ *hybridization with *cyp26 *(A,D,G,J,M,P,S,V,Y) or *hoxb1b *(B,E,H,K,NQ,T,W,Z) probes or double *in situ *hybridization with both probes (C,F,I,L,O,R,U,X,A') is shown for wild-type embryos (A-C), untreated *ich *embryos (D-F), or *ich *embryos treated with bmp2bMO (G-I), the two β-cat MOs (J-L), bmp2bMO plus the two β-cat MOs (M-O), or *ich *embryos injected with *antivin *mRNA (P-R), *antivin *mRNA plus bmp2bMO (S-U), SU5402 (V-X), or SU5402 plus bmp2bMO (Y-A'). Wild-type embryos [A-C] are shown in dorsal views, while *ich *embryos are shown in lateral views. The neuroectodermal and tailbud expression domains of *cyp26 *in wild-type embryos are marked with arrow and star, respectively. All embryos are at ~100% epiboly.

At 10 hpf, *cyp26, hoxb1b, otx1*, and *gbx1 *clearly mark distinct neurectodermal territories in wild-type embryos: *cyp26 *is expressed both in the anterior neurectoderm and in the most posterior region of the embryo (Figure 3A,C, arrow and star, respectively) [45], *hoxb1b *marks neuroectoderm posterior to the prospective rhombomere 3/4 boundary (Figure 3B,C) [46]. In *ich *embryos, as expected [7], expression of *otx1, hoxb1b, gbx1*, and the anterior domain of *cyp26*, is absent (Figures 3D-F). Only the posterior domain of *cyp26 *is still expressed (Figure 3D), and its expansion is consistent with the observation that the most posterior neurectoderm still forms in *ich *embryos [47].

When injection of bmp2bMO is used to inhibit BMP signaling in these embryos, all four neurectodermal markers are expressed robustly in correct relative AP order in approximately the same AP position as in wild-type embryos (Figures 3G-I). The width of the expression domains is almost identical to those of wild-type embryos, but the expression is radial, extending completely around the embryo, rather than restricted to the dorsal side. (Similar results were observed with two other markers, *otx1*, expressed in the prospective forebrain and midbrain [48], and *gbx1*, expressed from the midbrain/hindbrain boundary posterior towards rhombomere 2 [49] (Additional file 1, Figure S1A-I)).

When βcat1MO + βcat2MO injection is used to inhibit BMP signaling, the results are similar in that all four markers are expressed in correct AP order (Figure 3J-L, Additional file 1, Figure S1J-L). The major difference is that expression is shifted towards the posterior of the embryo. The anterior *cyp26 *domain and the *otx1 *and *gbx *domains are wider, and the area of *hoxb1b *is restricted to a more posterior region of the embryo than is the case in wild-type or in bmp2bMO-injected *ich *embryos. We also tested the effects of injecting all three MOs on expression of the four markers (Figure 3M-O, Additional file 1, Figure S1M-O). Results for the more posterior markers *gbx1 *and *hoxb1b *are similar to the embryos treated with the two βcatMOs alone, while the expression of *otx1 *and the anterior domain of *cyp26 *is expanded to encompass the whole anterior 60% of the neurectoderm. Treatment with bmp2bMO, but not with the two βcatMOs, eliminates BMP signaling in the animal pole region (Figure 1C-E), thus permitting expression of these anterior markers in the former, but not latter, embryos. The expansion of expression of the anterior markers toward the vegetal pole in the triple MO-treated embryos, compared to embryos treated with bmp2bMO alone, is evidence for a posteriorizing role of Wnt/β-catenin signaling. When this signaling is eliminated by MO treatment, there is a marked posterior expansion of the zone of anterior identity.

**Figure 4:**
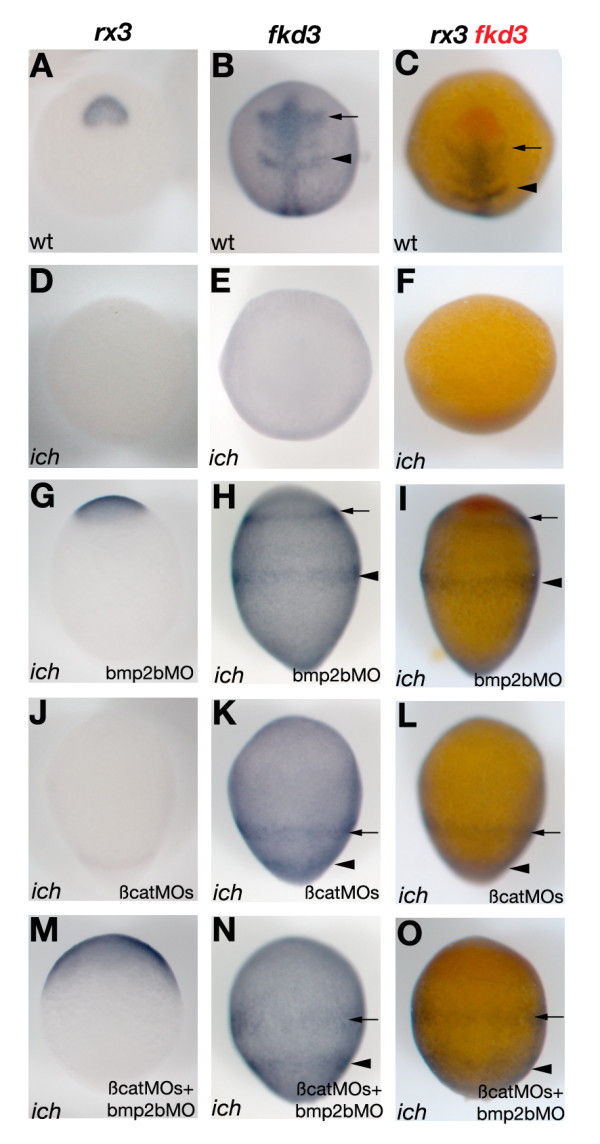
**AP neurectodermal patterning is retained at 11 hpf in embryos lacking BMP signaling and an active organizer**. Single *in situ *hybridization with *rx3 *(A,D,G,J,M) or *fkd3 *(B,E,H,K,N) probes or double *in situ *hybridization with both probes (C,F,I,L,O) is shown for wild-type embryos (A-C), untreated *ich *embryos (D-F), or *ich *embryos treated with bmp2bMO (G-I), the two β-cat MOs (J-L), or bmp2bMO plus the two β-cat MOs (M-O). In wild-type embryos, *rx3 *demarcates the eye-field (A,C) and *fkd3 *the diencephalon (arrows) and rhombomere 5 (arrowheads) of the hindbrain (B,C).

Neurectodermal markers expressed at later times in these treated embryos also show correct relative AP patterning (Figure 4). At 11 hpf, the wild-type expression of both the eye-field marker *rx3 *(Figure 4A,C) [50] and the diencephalic marker *fkd3 *(Figure 4B,C) [51] is completely absent in *ich *embryos (Figure 4D-F) but is conserved in the *bmp2*-morphant *ich *embryos (Figure 4G-I). These results indicate that the AP patterning of post-gastrula embryos is also independent of the presence of the organizer, as long as BMP signaling is absent. Identical results were obtained at this stage using the midbrain-hindbrain boundary (MHB) marker *pax2.1 *[52] and the hindbrain rhombomere 3 and 5 marker, *krox20 *[53] (data not shown). The 11 hpf 'ciuffo' embryos also show correct relative AP patterning of *fkd3 *(Figure 4K,L), *pax2.1*, and *krox20 *markers (data not shown). However, these embryos do not express *rx3 *(Figure 4J,L), the most anterior marker tested, presumably because of the active BMP signaling present at the animal pole.

A comparison of AP patterning in the neurectoderm of βcatMO-injected *ich *('ciuffo') embryos and *bmp2b *morphant embryos cannot easily be carried out at later stages. After 11 hpf, bmp2bMO-injected *ich *embryos, like *swr/bmp2b *embryos [30], begin to burst and die as a consequence of severe constriction movements. The 24 hpf βcat1MO-injected *ich *embryos do survive and we already had good indication that their neurectoderm exhibited correct patterning [8,23]. Two-color *in situ *hybridizations with four probes (*emx1, krox20, val, hoxb6b*) showing distinct restricted expression along the AP axis confirm and extend these results (Additional file 2, Figure S2). While none of these markers were expressed in *ich *embryos (Additional file 2, Figure S2B,E,H), they were all expressed in correct relative spatial order in embryos co-injected with βcat1MO and βcat2MO. The more anterior markers (*emx1 *in Additional file 2, Figure S2A-C', *krox20 *in Additional file 2, Figure S2D-I') were detected proximal to the yolk, while the posterior markers (*krox20 *in Additional file 2, Figure S2A-C', *val *in Additional file 2, Figure S2D-F' and *hoxb6b *in Additional file 2, Figure S2G-I') were expressed distal to the yolk. These results, along with the data presented on 10 hpf and 11 hpf embyos, indicate that the patterning pathways necessary to establish major neuroectodermal territories (forebrain, hindbrain and neural tube) are functional in 'ciuffo' embryos, and that these regions are demarcated with correct relative AP pattern.

We also tested *ich *embryos injected with βcatMO1 or bmp2bMO for expression of the more posterior *hox *genes *hoxb6b*, *hoxb8a*, *hoxa9a*, *hoxd12*, and *hoxc13a *at 24 hpf. (The majority of bmp2bMO-injected embryos die during earlier stages of development, but ~ 2% survive to 24 hpf.) Although we were unable to obtain good signals by *in situ *hybridization, and thus, were not able to determine if these genes were expressed in a proper relative AP pattern, RT-PCR assays did indicate the relative level of their expression in these embryos (Additional file 3, Figure S3). A signal was obtained for *hoxb6b*, *hoxb8a*, *hoxa9a *and *hoxd12*, but not for *hoxc13a*, in βcatMO1-injected *ich *embryos (Additional file 3, Figure S3 - lane 3), and for all five of these genes in bmp2bMO-injected *ich *embryos (Additional file 3, Figure S3 - lane 8). These results show that embryos lacking both organizer and BMP signaling have the potential to express not only anterior neurectodermal markers, but also trunk posterior markers. We also tested for expression of these genes in untreated *ich *embryos (Additional file 3, Figure S3 - lane 2) and found that the four most posterior markers were robustly expressed. This result is consistent with the finding that *ich *mutant embryos do express the earlier posterior neurectodermal markers *sox3 *and *zic2.2*, which are involved in tail neural tube formation [47].

### AP patterning in the absence of the organizer is modulated by the same factors as in wild-type embryos

Wnt, Nodal, and FGF signaling pathways are all known to be involved in the posteriorization of neural tissue [45,54-56], an effect they achieve by epistatic interactions between themselves and the retinoic acid (RA) pathway [45,57]. As noted above, the absence of canonical Wnt/β-catenin activity in *ich *embryos injected with bmp2bMO expands *cyp26 *and *otx1*-expressing anterior neurectoderm towards the vegetal pole in comparison with embryos treated with bmp2bMO alone (Figure 3G,J,M; Additional file 1, Figure S1G,J,M). The posterior expansion can also be seen in the comparison of *rx3 *expression in *ich *embryos injected with the three MOs and embryos injected solely with bmp2bMO (Figure 4G,M). Also consistent with a posteriorizing role for Wnt signaling is the finding that the more posterior neurectoderm, as indicated by regions of *hoxb1b, gbx1*, and *fkd3 *expression, is shifted markedly to a more vegetal position when *ich *embryos are treated either with βcatMOs alone or with βcatMOs + bmp2bMO compared to embryos injected with bmp2bMO alone (Figure 3H,K,N; Additional file 1, Figure S1H,K,N; Figure 4H,K,N). These results show that the posteriorizing role of Wnt/β-catenin signaling is completely independent of BMP signaling. That there is still ample expression of the hindbrain markers *hoxb1b *and *gbx1 *in *ich *embryos injected with both βcatMOs suggests that other factors must act independently (or perhaps upstream of Wnt8 signaling) as posteriorizing agents. Supporting this idea is that 'ciuffo' embryos still express at least four posterior hox genes (Additional file 3, Figure S3).

TGFβ proteins also act as posteriorizing factors during normal zebrafish development. Injection into wild-type embryos of mRNA for *antivin*, a potent antagonist of Nodals and Activins, results in dramatic anteriorization, with the most severely affected embryos losing all parts of the neural tube except telencephalic tissue [54]. To determine if ligands inhibited by antivin are posteriorizing factors in the absence of BMP signaling and organizer, we co-injected *antivin *mRNA and bmp2bMO into *ich *embryos. These embryos exhibited complete anteriorization of the neuroectoderm, as indicted by ubiquitous expression of the anterior markers *cyp26 *and *otx1 *(compare Figure 3G with 3S; Additional file 1, Figure S1G with S1S) and complete loss of the more posterior markers *otx1 *and *gbx1 *(compare Figure 3H with 3T; Additional file 1, Figure S1H with S1T). The lack of expression of the anterior markers at the vegetal pole itself is not due to an absence of expression in vegetal neurectoderm; rather, it is a consequence of a failure to complete gastrulation by those embryos co-injected with both *antivin *mRNA and bmp2bMO (arrowheads in Figure 3S-U and Additional file 1, Figure S1S-U indicate the vegetal extent of the germ-ring at 10 hpf), an observation consistent with a role of *activin *in gastrulation movements shown in *Xenopus *[58,59]. We also used an alternative method of inhibiting TGFβ signaling, the application of SB431542, a compound that inhibits Smad2/3-mediated TGFβ-signaling [60]. Embryos grown in SB431542 also exhibited a concentration-dependent anteriorization, and also stalled during gastrulation (Additional file 1, Figure S4). Interestingly, the posteriorizing effects appear not to be entirely due to Nodal signals, as inhibiting expression of these ligands by co-injection of MOs against *squint *(*sqt*) and *cyclops *(*cyc*), the two zebrafish Nodal homologues known to be expressed during these gastrulation stages [61-64], showed much less posterior expansion of *cyp26 *expression compared to *antivin *treatment (compare Figures 2G,S to Additional file 4, Figure S4M) and dramatically less reduction of *hoxb1b *expression (compare Figure 3H,T and Additional file 4, Figure S4N).

FGF signaling is yet a third signal transduction pathway known to posteriorize neurectoderm in wild-type embryos [16,45,65,66]. To check if this signaling pathway also functions in embryos devoid of organizer and BMP signaling, we treated *ich *embryos inhibited in BMP signaling with SU5402, a small molecule inhibitor of FGF receptor activity [67]. Treatment with this compound caused the expansion of *cyp26 *and *otx1 *anterior marker domains (compare Figure 3G with 3Y; Additional file 1, Figure S1G with S1Y) and elimination of expression of *hoxb1b *and *gbx1 *posterior markers (compare Figure 3H with 3Z; Additional file 1, Figure S1H with S1Z). However, as SU5402 treatment itself appears to reduce the intensity of *cyp26 *and *hoxb1b *expression, we also employed other methods of inhibiting FGF signaling (the reduction in staining intensity might be due to a role of early FGF signaling in neural capacitation [T. Kudoh, personal communication]). Injections of mRNAs encoding a dominant negative FGF receptor (XFD) [68] or the MAPK-pathway antagonist mkp3 [69] yielded a similar expansion of the *cyp26 *domain (Additional file 5, Figure S5M,S compared with Figure 4G), but was less effective in eliminating the *hoxb1b *domain (Additional file 5, Figure S5N,T compared with Figure 4H). These latter results, however, are complicated by obvious effects on gastrulation movements that result in asymmetry of staining around the circumference of the embryo. All three FGF signaling inhibitors do, however, have dramatic anteriorizing effects on expression of neurectodermal markers.

In summary these observations show not only that embryos that fail to exhibit an early dorsal organizer show correct relative AP patterning in the absence of BMP signaling, but that this patterning is under posteriorizing control of Wnt-, TGFβ-, and FGF-signaling, just as is the case in wild-type embryos.

### Diffuse neurectoderm surrounds mesoderm and endoderm in embryos lacking canonical Wnt signaling and impaired in BMP signaling

**Figure 5:**
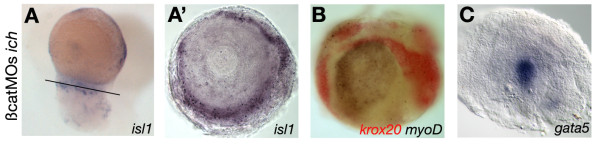
**Germ layer segregation in 'ciuffo' embryos**. Sections through the posterior protrusions of 24hpf 'ciuffo' embryos stained with characteristic germ layer markers show that neuroectoderm specific markers. *islet1 *(A) and *krox20 *(B) are expressed in the outermost layer of the protrusion. The endodermal marker *gata5 *(C) is expressed in the innermost layers, whereas the mesodermal marker *myoD *(B) can be observed in between. Sections were made of 24 hpf *ich *embryos that had been injected with βcatMO1 and βcatMO2, hybridized as whole mounts with the indicated probes.

As we have shown above, 24 hpf 'ciuffo' embryos are characterized by a protrusion of tissue, extending away from the yolk, that expresses a set of neurectodermal genes in proper AP order. The extension arises from abnormal and excessive epiboly movements, which causes tissue to extend far beyond the normal limit of migration, the vegetal pole of the yolk. At 24 hpf, as a consequence of this morphogenetic movement, a protruding tissue can be observed at the posterior edge of the yolk at the site of the earlier blastopore closure. As both neuroectodermal and mesodermal markers are expressed in this protrusion with overlapping patterns [8], we tested whether cells derived from each germ layer still segregate together, or not. Sections through the 'ciuffo' protrusions revealed that neuroectodermal markers (*isl1, krox20*) are expressed in the outer layers (Figure 5A,B), the mesodermal marker *myoD *in the medial layers (Figure 5B), and the endodermal marker *gata5 *is expressed in the innermost layer (Figure 5C). Thus, the segregation of germ layers is preserved in the protrusion of 'ciuffo' embryos. Expression of *isl1 *in the outer layer (Figure 5A) shows that this layer contains cells with neuronal identity, but that rather than being organized within a neural tube, they are located diffusely in a sheath of tissue that surrounds mesodermal and endodermal derivatives. Thus, when canonical Wnt signaling is absent and BMP signaling is highly reduced, the neurectoderm retains its AP pattern and can form cells with neuronal identity, but the overall organization of this tissue is quite different from the vertebrate embryo neural tube.

## Discussion

### Correct anteroposterior patterning in the absence of the organizer and BMP signaling

The work described here adds to extensive evidence suggesting that an early dorsal organizer is not required for proper AP patterning of the vertebrate embryo. Surgical removal of the organizer in mouse, *Xenopus*, chick, and fish [9,11-13,70], transplantation experiments in zebrafish [16,71], and genetic "ablations" of organizer tissue in mouse [10,14] and zebrafish [15,17,72] all strongly suggested that the organizer is not required for generation of AP pattern.

However, these earlier experiments were not definitive. The ablation experiments in chick and mice were often followed by an almost complete restoration of the organizer [11], or did not completely eliminate the expression of all organizer-related genes [13]. In the zebrafish embryo, most genes with organizer activity are expressed before the shield becomes visible; thus, physical removal of this structure can not eliminate organizer activity completely [12]. Zebrafish embryos impaired in the Nodal signaling pathway display defective shield formation [15,17], but dorsal expression of several organizer markers (e.g. *chd*, *gsc*, *boz*) can be observed during the development of mutant embryos [17,73], and axis formation is not completely abolished even in *sqt;cyc;boz *triple mutants [72].

In the zebrafish embryo, a true genetic ablation of the dorsal organizer resulting in failure to induce any dorsal markers is really only achieved by the severe reduction of maternal β-catenin-2 in mutant *ich *embryos, or in embryos treated with MOs against *β-catenin-2 *[7,8]. However, as *ich *embryos fail to form head, trunk, and most neurectodermal tissues, their phenotype is not informative for determining if loss of dorsal organizer results in altered A-P pattern.

The lack of importance of the organizer in generating neurectodermal A-P pattern dramatically revealed only when BMP signaling is inhibited in embryos lacking the organizer. The first indication of this was the outcome of an experiment of Ober and Schulte-Merker [18] in which vegetal yolk was removed from wild-type zebrafish embryos just after fertilization. Such embryos were completely ventralized with lack of organizer (embryonic shield) and neurectoderm formation, indicating that determinants of organizer formation are localized at or near the vegetal pole. However, when this vegetal yolk removal was performed on *swr/bmp2b *embryos (which lack BMP signaling) neural tissue did form and at least two neural markers appeared with appropriate AP pattern, but were expressed radially. A similar result was obtained by comparing double *boz;chd *embryos, unable to form an organizer and neurectoderm, with triple *boz;chd;swr *embryos, in which the ectoderm is neuralized and markers of the midbrain hindbrain boundary and rhombomeres are correctly patterned [74]. In the experimental work we have presented above, we can explain the restoration of correct A-P neurectodermal pattern found in embryos inhibited in expression of both *β-catenins *[8] as a consequence of the inhibition of BMP signaling due to the massive ectopic expression of *chd *in these embryos [23]. Our results show that this patterning is equivalent to embryos lacking maternal β-catenin-2 that have been inhibited in BMP signaling by injection with *bmp2b*MO.

We also provide the first report that simultaneous inhibition of BMP signaling and organizer formation results in proper neurectodermal patterning even during gastrula stages. In *Xenopus*, neurectodermal markers were expressed radially in proper relative AP order in the brain of embryos lacking both organizer and BMP signaling was shown at later neurula stages, but not during gastrulation [19]. In this case, the knockdown of BMP signaling was obtained by administration of MOs against three BMPs and organizer functions were eliminated by UV treatment or administration of a β-catenin MO. Thus, the consequence of simultaneous elimination of both BMP signaling and organizer formation in both zebrafish and *Xenopus *is robust formation of radially organized neurectoderm, including all regions of the brain, with proper relative AP pattern.

It is interesting that the pattern of embryonic neurectodermal AP domains is highly conserved between chordates and the radially organized hemichordates [75,76], suggesting that the deuterostome ancestor had the same AP pattern and that generation of the AP axis is independent of the organizer. Moreover, the independence of the AP and DV axis, previously recognized in hemichordates [77], is also clearly shown in the zebrafish embryo when dorsal organizer formation is eliminated along with a reduction in BMP signaling.

### Multiple organizer-independent signaling pathways posteriorize the neurectoderm

**Figure 6:**
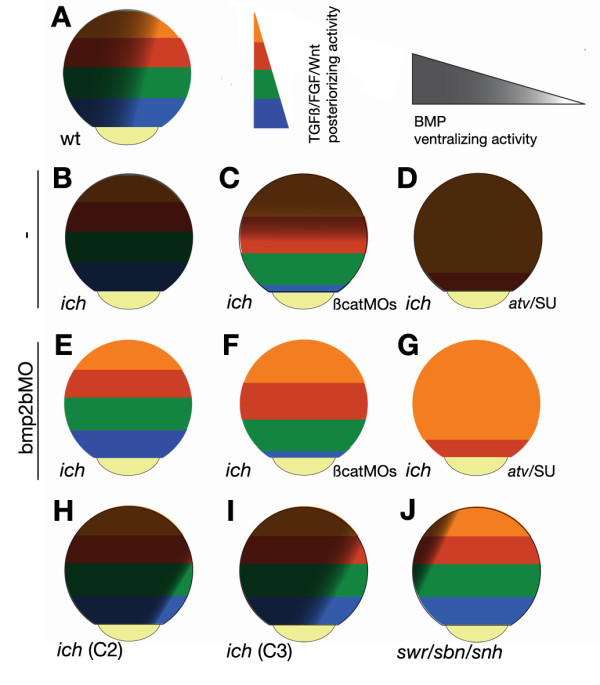
**Patterning of the zebrafish neurectoderm is achieved by the parallel action of posteriorizing "transformative" signals and "activating" BMP-antagonists**. (A-J) Neurectodermal regions are color coded as indicated with the most anterior territory represented in orange and the most posterior in blue. The BMP signaling gradient is indicated by grey shading, so that a brownish shading superimposed on the colored regions represent areas of the embryo unable to form neurectoderm due to the absence of an "activating" signal. Embryos of indicated genotype and treatment are diagrammed at 80-90% epiboly. See text for details.

As presented in the Results section, Wnt, Nodal, and FGF signal transduction pathways, operating in the zebrafish germ-ring, posteriorize the neurectoderm in an organizer-independent manner. These findings are completely supportive of the "two-step model" of Nieuwkoop [78], with the "activating," anti-BMP signals originating from the organizer, and the "transformative" posteriorizing signals emanating from the germ-ring (Figure 6A).

It is well established that one of the major posteriorizing signals during vertebrate embryogenesis is the canonical Wnt pathway, which acts as a morphogen along the AP axis of the neural plate to regulate the expression of patterning genes [55]. Zebrafish mutants which upregulate the pathway, such as *masterblind*/*axin1 *(*mbl*) [79] or *headless*/*tcf3 (hdl*) [80] exhibit a loss of anterior neural structures, whereas impairment of Wnt signaling results in the expansion of anterior neural compartments [45,56]. Our results provide evidence that this posteriorizing effect of Wnt signaling is independent of the dorsal organizer. Injection of bmp2bMO alone into *ich *embryos clearly does not result in formation of organizer tissue (Figure 2C,F). Yet, inhibition of all detectable Wnt-signaling by the additional co-injection of the two β-catMOs [23] results in a marked expansion of anterior neuroectoderm (Figure 2M,O, Figure 3M,O). It should be noted as well that the persistence of considerable amounts of posterior markers under these conditions (Figure 2N, 3N) suggests that Wnts are not the only posteriorizing signals and that other, parallel-acting pathways function as well.

FGFs and activin-type TGFβ signals have also been implicated in AP patterning [54,65]. Accordingly, injection of inhibitors of FGF and Nodal/Activin signaling into zebrafish embryos results in dramatic anteriorization of neural tissue [16,45,54]. By blocking these pathways in bmp2bMO-injected *ich *embryos, we were also able to induce dramatic reduction in posterior neuroectoderm and expansion of anterior neuroectodermal markers (Figure 3S-U,3Y-A', Additional file 1, Figure S1S-U,Y-A').

An alternative explanation for the observed shift in the position of the posterior markers in embryos treated with β-catMOs might be an altered posterior movement of mesodermal cells. By regulating cell-cell adhesion, the BMP gradient across the DV axis controls convergence and extension movements of lateral mesodermal cells in wild type embryos [81]. In 'ciuffo' embryos, we have demonstrated that an ectopic gradient forms between cells with active BMP signaling at the animal pole and the *chd *expressing cells of the germ-ring (Figure 1). This gradient is likely responsible for the excessive migration of cells towards the vegetal pole and the formation of the characteristic protrusions at 24 hpf. However, as triple morphant (bmp2bMO and the two β-catMOs) embryos show an almost identical posteriorization as 'ciuffo' embryos, without any sign of abnormal posterior cell movements, we think it unlikely that the posteriorization in 'ciuffo' embryos is due to such movements. We followed the post-gastrulation development of untreated and morphant *ich *embryos and although excess tissue can be readily observed at the vegetal pole of 'ciuffo' embryos at 12.5-13.5 hpf, the triple morphants resemble *ich *embryos treated with bmp2bMO alone, with tissue accumulation at both animal and vegetal sides (Additional file 6, Figure S6).

The "two step model" provides a comprehensive explanation for our observations (Figure 6). When the "activating", BMP-antagonist signals are absent, the effects of the "transformative" signals, although present, are masked by the lack of the neuroectoderm. This is the case in *ich *embryos, which lack expression of BMP antagonists and in which presence of BMPs is ubiquitous (Figure 6B). In contrast, the removal of BMP-signaling transforms the complete ectoderm to neuroectodermal fate, and patterning domains in *ich *embryos appear as circumferential rings (Figure 6E). The normal "transformative" posteriorizing signals clearly operate in these embryos but are not restricted by the ventral cues that would have functioned in the wild-type state. Under such conditions an impairment in the level of posteriorizing factors can be easily observed as a vegetal "shift" (anteriorization) in the position of neuroectodermal expression domains in *ich *embryos. The shift can be caused by inhibition of germ-ring Wnt8 signaling by treatment with both β-catMOs (Figure 6F), or by inhibition of Nodal/Activin with antivin or SB431542, or by inhibition of FGF signaling with SU5402 or mkp3 or XFD (Figure 6G). However, in the case of *ich *embryos treated with these Nodal/Activin or FGF inhibitors, the anteriorization is only observed in embryos also inhibited in BMP signaling (Figure 6G) as, otherwise, the ubiquitous expression of BMPs prevents formation of any neurectoderm at all (Figure 6D). 'Ciuffo' (i.e., βcat1MO-treated *ich*) embryos (Figure 6C) present a state quite similar to bmp2bMO-treated *ich *embyros, as the lack of canonical Wnt signaling induces large amounts of BMP-antagonists in a wide band extending from the germ-ring, allowing the "transformative" Nodal/Activin and FGF signals to operate. However, such embryos still retain BMP signaling in the animal pole region of the embryos and thus, neurectodermal markers are not expressed in the animal-most fourth of the embryo.

This model (which is similar to the one proposed earlier by Meinhardt based on theoretical considerations [5], and more recently by Niehrs based on the review of the existing literature [82]) also offers an explanation for multiple aspects of the dorsalized and ventralized phenotypes described in several zebrafish mutants. For example, the progressive loss of anterior neural tissue observed in embryos with different degrees of ventralization, such as less severe *ich *embryos (C2,C3,C4) [7] or *chd *and *nog1 *double deficient embryos (CI,CII) [83], is due to the smaller size of the ''activation" domain and not to a change in the "transformative" signal (Figure 6H,I). As anti-BMP signals fail to reach the animal-most region of the gastrula, markers of the forebrain (and often midbrain) will not be turned on. Similarly, in mutants of the BMP pathway, such as *swr, somitabun *(*sbn*) and *snailhouse *(*snh*) [30], the oversized BMP-absent area results in laterally expanded, but otherwise correctly positioned, expression domains during gastrulation (Figure 4J) [32,84], indicating that the "transformative" signal is not perturbed in such embryos.

Our observations clearly refute the argument that the zebrafish AP and "classical" DV axes are equivalent [20,21], as it is clear from work presented here that the prospective AP axis of the embryo coincides with the early animal-vegetal axis of the zygote. As *Xenopus *embryos deficient both in organizer formation and BMP signaling also express AP patterning markers circumferentially [19], we propose that the concordance of the AP and animal-vegetal axis is a general feature of the anamniote embryos.

### 'Ciuffo' embryos may reveal ancestral anteroposterior radial neurectoderm patterning

Two striking features of the neurectoderm of 24 hr 'ciuffo' embryos are the radial organization along the full AP extent of the embryo, and the location of cells of neuronal identity in a diffuse network close to the outer surface, while tissues of mesodermal and endodermal identity reside within the embryo. This organization is reminiscent of the supposed pre-Urbilaterian, proto-Eumetazoan ancestor. Comparative studies suggest that the Eumetazoan ancestor had a diffuse nerve net [85] and members of Cnidaria, the sister clade of Eumetazoa, still possess such a primitive nervous system. Although it is yet unclear how exactly the patterning of this structure occurs, it has been suggested that FGF and TGFβ signaling pathways play an active role in specifying the neuronal identities observed in *Nematostella vectensis*, an anthozoan cnidarian [86]. This view coincides with the evolutionary scenario suggested by Arendt et al. [87] in which BMP signaling was originally involved in the patterning of neuronal cell types and it was only adapted later to control formation of a centralized nervous system. Indeed, in several anthozoan species, asymmetric expression of BMPs and BMP antagonists has been observed, suggesting both that the origins of the bilaterian DV patterning system predate the Cnidarian - Eumetazoan split and that the system was originally not involved in driving nervous system centralization [88-91]. Strikingly, in *Nematostella *most BMP-components are expressed in the endoderm [90] and recent functional data indicates a major role in endoderm patterning (and a lesser role in neural differentiation) for the BMP pathway [92]. It is noteworthy that *chordin *and different *bmp/dpp *genes are expressed on the same side of the directive axis in *Nematostella*, suggesting a patterning center with functional similarities to the *chd/admp*-expressing vertebrate organizer [92]. Such a center alone is sufficient to create a BMP gradient in the embryo [93], most likely through the shuttling of Chd/BMP complexes [94]. As a similar system was recently described for sea urchins as well [95], we suggest that this might constitute a prototypical BMP signaling paradigm, which later evolved into the scalable BMP signaling system observed in some Bilaterians.

Interestingly, some larval hemichordates (which together with echinoderms form the sister group of vertebrates in Deuterostoma) also possess a diffuse, epidermal neural network (reviewed in Gerhart et al. [96]). New research, however, suggests that this feature is not homologous with the vertebrate neural plate (as has been previously suggested [77]), but is a transient larval adaptation of certain hemichordate species [97]. As whenever a CNS is present in Bilateria, it develops on the side of BMP antagonism [87], it can be inferred that a centralized nervous system, regulated by BMP signals, is the ancestral state for all bilaterians. In echinoderms and certain hemichordates, where centralization is not observed, a secondary loss might have occurred during the evolution of these lineages [98]. By eliminating Wnt signaling and greatly reducing BMP signaling in 'ciuffo' embryos, we can observe the lack of restriction of neurectoderm to a particular DV level. That the potential for such a "diffuse nerve net" exists in a chordate species suggests that the evolutionary transition between a centralized and diffuse nervous system might have involved only a quite limited number of steps.

The organizer apparently arose in a chordate ancestor, as a signaling center homologous to the vertebrate organizer has been found in the cephalochordate (amphioxus) *Branchiostoma floridae *[99]. As tunicates are now considered the sister group of vertebrates, and amphioxus is more distantly related, it is likely that the organizer was lost in tunicates [99]. Chordate embryos have evolved the organizer to provide among other signaling functions, a specialized temporal and spatial program of BMP antagonist expression which acts on a pre-existing, extremely highly conserved pattern of AP tissue specification. As the AP pattern of gene expression appears to be widely conserved throughout the animal kingdom, it will be of interest to test whether the same pattern-generating "transformative" signals operate in hemichordates and non-vertebrate chordates. Wnt signals have already been shown in cephalochordates (amphioxus) to be expressed posteriorly, around the germ-ring, very much as in fish and amphibians, indicating that Wnts are a posterior "transformative'" signal characteristic of chordates [99-101]. In amphioxus, the effects of this signal appear to be strongest at the posterior end of the embryo, while retinoic acid has a more important role in determining AP identity elsewhere [100]. Wnt signaling is an extremely ancient mechanism of patterning the body axis [82,102], as it operates to specify position along the main body axis of the cnidarian *Nematostella vectensis *(a sea anemone) [103,104]. There is virtually nothing known, however, about FGF and Nodal signaling as potential "transformative" signals in non-vertebrate embryos.

## Conclusions

Our work provides evidence for the organizer-independent AP patterning of the neuroectoderm in the developing zebrafish gastrula. We observed correctly located AP neurectodermal domains in the organizer-less *ich *mutant embryos when BMP-signaling was inhibited. The position and size of these domains depends mainly on the action of Wnt-, FGF- and Nodal signaling, originating from the germ-ring of the gastrula. These observations can be easily interpreted within the framework of Nieuwkoop's "two step model": the observed neurectodermal pattern in wild type fish is the result of the concerted action of "activating" and "transformative" signals (in the case of the zebrafish, BMP-antagonists and germ-ring-derived morphogens, respectively).

Our results also clearly refute recent proposals about the equivalency of the AP and "classical" DV axes in anamniotes, as the concordance of the AP axis with the AnVeg axis of the early embryo is evident.

When both Wnt- and BMP-signaling was inhibited in *ich *embryos, they developed into a well patterned tube-like structure, where a neurectodermal sheet envelopes inner mesodermal and endodermal tissues. In this neurectodermal domain scattered neuronal progenitors can be detected. Therefore, we speculate that vertebrates still retain the genetic program to form an ancient radially-organized diffuse neural net, and that only a limited number of changes in this program may have been necessary to form a neural-tube type of organization.

## Methods

### Zebrafish strains and husbandry

Zebrafish were maintained under standard conditions [105]. Wild-type embryos were derived from AB parents, while *ichabod^p1^*(*ich*) embryos were obtained by breeding homozygous *ich *females with *brass *or *ich *males. Only those *ich *females that reproducibly yielded severely ventralized Class 1 (or Class 1a) phenotypes (for details see [7,40]) were used. All animal work described here was carried out under Protocols 700433 and 801973 approved by the Institutional Animal Care and Use Committee (IACUC) of the University of Pennsylvania. This committee approves animal work only if it follows internationally recognized ethical and experimental guidelines.

### Morpholino antisense oligonucleotide and mRNA injections

β-catenin-1-MO (βcat1MO) [8], β-catenin-2-MO (βcat2MO) [8], bmp2bMO [26], sqtMO [106], and cycMO [107] were manufactured by Gene Tools (Philomath, OR) and the sequences of each are as published. Clones used to prepare sense mRNA were as follows: *mkp3 *in pCS2+ [69] and *antivin *(*lefty1*) in pCS2+ [108]. mRNAs were synthesized and capped using an mMessage mMachine Kit (Ambion), following the manufacturer's protocol. RNAs and morpholinos were stored in dH_2_O at -20°C and injection solutions were prepared by diluting a 2 × stock of reagent in dH_2_O with an equal volume of Dulbecco's modified phosphate-buffered saline containing 5% phenol red (Sigma). Approximately 1 nl of solution was injected into the yolk at the base of blastomeres of 1-4 cell embryos. The concentration of these antisense and sense reagents that were injected per embryo were as follows: βcat1MO and βcat2MO (β catMOs) (3 mM each), bmp2bMO (0.4 mM), sqtMO and cycMO (0.6 mM each), and *antivin *(*atv*) mRNA (200 ng/μl).

### Small molecule treatments

SB431542 (Sigma) and SU5402 (Calbiochem) were used at indicated concentrations to block TGF β-activin and FGF signaling, respectively. The reagents were dissolved in DMSO and added to the culture medium at the 1000-cell stage. Embryos were kept in the dark until fixed.

### RT-PCR experiments

Total RNA isolated from pooled samples of 10 embryos was used to generate cDNA using the SuperScript II kit (Invitrogen), following the manufacturer's protocol. The primers used in the experiment were as follows (all shown in 5'-3' orientation): krox20-F: CTGCCAGCCTCTGTGACTA, krox20-R: CCATGGTGCAGCTGAGAGT, hoxb6b-F: CTGACCGCTCGTGCGCTAT, hoxb6b-R: ATCTTCCTCATCGCTGACCTT, hoxb8a-F: GCAGAGTCCATGTGCGGTAA, hoxb8a-R: CAATCCGACGCTTGCGTGTT, hoxa9a-F: AACTGAGCCACCGACGGTTA, hoxa9a-R: TCTTCATCCTGCGGTTTTGGA, hoxd12-F: GTCACTGAGCGCCCAGAAT, hoxd12-R: CAGTTCCAATCTGTCCGAAA, hoxc13a-F: CCACGTCACGATGCATTGAT, hoxc13a-R: TAACTTGACGTTCTGAGAGGTT, ef1a-F: ACCGGCCATCTGATCTACAA and ef1a-R: CAATGGTGATACCACGCTCA.

### Whole mount *in situ *hybridizations and immunohistochemistry

The following clones were used to prepare antisense probes for hybridization: *p63 *[35], *krox20 *[53], *emx1 *[109], *val *(D. Grunwald, cDNA library), *cyp26 *[45], *hoxb1b *[46], *hoxb6b *[110], *otx1 *[48], *gbx1 *[49], *rx3 *[50], *boz *[38], *gsc *[25], *chd *[111] and *fkd3 *[51]. Antisense RNA probes were synthesized and two color *in situ *hybridization was carried out as previously described [23]. For embryos older than 24 hpf (Figure 5; Additional file 1, Figure S1) NBT/BCIP was used as primary chromogen, as it gave better results. Whole mount embryos were imaged with a Leica MZ12 stereomicroscope, using a Roper Scientific Photometrics RGB Vision MS-C digital camera system (CRI, Inc, Boston, MA).

For manual sections (Figure 5) embryos subjected to whole-mount *in situ *hybridisation were cleared in serial incubations of glycerol (25, 50, 75 and 95%). Sections were placed in a drop of glycerol, cover-slipped, and imaged with 40 × (0.8 NA) water-immersion lens using a Nikon E1000 microscope connected to a digital camera (Jenoptik) operated by Openlab (Improvision) software.

For immunohistochemistry, embryos were fixed in 4% PFA, blocked in standardized blocking solution (10% fetal bovine serum, 1% DMSO, 0.8% Triton-X in PBS), incubated overnight at 4 C° with a 1:100 dilution of anti-phospho-Smad1/5/8 antibody (Cell Signaling Technology), followed by a 1:200 dilution of goat anti-rabbit Alexa Fluor 488-conjugated antibody (Invitrogen). To visualize the nuclei, embryos were incubated in 1:1000 dilution of TO-PRO^®^-3 iodide (Invitrogen) DNA stain in PBST. After several washes the specimens were mounted in 1% low melting point agarose, and imaged on a Leica TCS SP Confocal Microscope using ×10 objectives. Image reconstruction was performed using Volocity (Improvison) software.

All figures were composed using Adobe Photoshop and Illustrator (CS3).

## Authors' contributions

MV conducted the experimental design and execution, and drafted the manuscript. SM performed some of the morpholino injection and *in situ *hybridization experiments. ESW oversaw the design of the experiments and the writing the final draft the manuscript. All authors read and approved the final manuscript.

## Supplementary Material

Additional file 1 **Figure S1 - Wnt-, TGFβ- and FGF-signaling have pivotal roles in the posteriorization of the neuroectoderm of embryos both inhibited in BMP signaling and devoid of organizer activity**. Single *in situ *hybridization with *otx1 *(A,D,G,J,M,P,S,V,Y) or *gbx1 *(B,E,H,K,Q,T,W,Z) probes or double *in situ *hybridization with both probes (C,F,I,L,O,R,U,X,A') is shown for wild-type embryos (A-C), untreated *ich *embryos (D-F), or *ich *embryos treated with bmp2bMO (G-I), the two β-cat MOs (J-L), bmp2bMO plus the two β-cat MOs (M-O), or *ich *embryos injected with *antivin *mRNA (P-R), *antivin *mRNA plus bmp2bMO (S-U), SU5402 (V-X), or SU5402 plus bmp2bMO (Y-A'). Wild-type embryos [A-C] are shown in dorsal views, while *ich *embryos are shown in lateral views. All embryos are at ~100% epiboly. Arrowheads in the *antivin *(*atv*) treated embryos point to the edge of the germ-ring. Click here for file

Additional file 2 **Figure S2 - Complete repression of β-catenin signaling in *ich *embryos induces neuroectoderm with correct AP pattern**. Untreated *ich *embryos do not express *emx1, krox20, val*, or *hoxb6b *(B,E,H), while their siblings coinjected with βcatMOs (C,C',F,F',I,I') express these neuroectodermal markers in a correct order at 22 hpf, with the anterior to posterior direction corresponding to proximal to distal relative to the yolk (compare A with C,C', D with F, F', and G with I,I'). The following probe-pairs were used: *emx1 *(blue) and *krox20 *(red) (A-C'), *krox20 *(red) and *val *(blue) (D-F'), and *krox20 *(red) and *hoxb6b *(blue) (G-I'). Click here for file

Additional file 3 **Figure S3 - Inhibition of Wnt- or BMP signaling in *ich *embryos induces typical anterior neurectodermal markers**. RT-PCR amplification of characteristic neurectodermal patterning markers (*krox20*, *hoxb6b, hoxb8a, hoxa9a, hoxd12, hoxc13a*) was carried out using oligo dT-primed cDNA samples from wild-type embryos (lanes 1-4), uninjected *ich *embryos (lanes 2,5,7,9), or *ich *embryos injected with βcat1MO ('ciuffo') (lanes 3,6) or bmp2bMO embryos (lanes 8,10). Whereas in *ich *embryos, only the posterior-most hox markers are present (lanes 2,7), 'ciuffo' embryos expressed almost the complete range of neurectodermal patterning markers examined, except that expression of *hoxc13a*, the most posterior marker examined, is absent, and the level of *hoxd12a is reduced *(lane 3). In contrast, bmp2bMO injection induced expression of all assayed neurectodermal markers (lane 8). Controls lacking RT were performed to make sure the signals observed were dependent on RNA (lanes 4-6,9,10). Click here for file

Additional file 4 **Figure S4 - Posteriorizing TGF**β **activity is only partly dependent on Nodal signals**. *In situ *hybridization with *cyp26 *(A,C,E,G,I,K,M) or *hoxb1b *(B,D,F,H,J,L,N) probes is shown for wild-type embryos (A,B), untreated *ich *embryos (C,D), *ich *embryos treated with bmp2bMO (E,F), SB431542 (G,H), sqtMO and cycMO (K,L), or bmp2bMO in combination with SB431542 (I,J) or sqtMO and cyc MO (M,N). A small molecular inhibitor of the TGFβ pathway, SB431542, has no effect on untreated *ich *embryos (G,H), but when it is applied to bmp2bMO injected *ich *embryos, it anteriorizes the neuroectoderm (I,J). These embryos have gastrulation defects; the arrowheads point to the position of the stalled germ-ring. Coinjection of sqtMO and cycMO with bmp2bMO results in a slight expansion of the *cyp26 *domain (M), and a mild reduction of the *hoxb1b *domain (N), showing that the posteriorizing effects of TGFβ signaling are dependent to some extent on Nodal-independent signals. Injection of sqtMO along with cycMO into untreated *ich *embryos has no effect on *cyp26 *and *hoxb1b *expression (K,L). SB431542 was used at 2.4 mM concentration; sqtMO and cycMO were 3 mM each. Wild-type embryos (A,B) are shown in a dorsal view, *ich *embryos (C-N) from a lateral view. All embryos are at ~10 hpf. Click here for file

Additional file 5 **Figure S5 - Antagonists of the FGF pathway can anteriorize *ich *embryos with impaired BMP signaling**. Single *in situ *hybridization with *cyp26 *(A,D,G,J,M,P,S) or *hoxb1b *(B,E,H,K,N,Q,T) probes or double *in situ *hybridization with both probes (C,F,I,L,O,R,U) is shown for wild-type embryos (A-C), untreated *ich *embryos (D-F), *ich *embryos treated with bmp2bMO (G-I), injected with *XFD *or *mkp3 *mRNAs alone (J-L and P-R), or in combination with bmp2bMO (M-O and S-U). The injection of mRNAs encoding a dominant negative FGF receptor, XFD, or a negative regulator of the MAPK pathway, mkp3, into bmp2bMO-injected embryos results in the posterior expansion of the anterior neurectodermal marker, *cyp26 *(M,O, and S,U, and a reduction of the posterior neurectodermal domain, marked by *hoxb1b *(N,O and T,U). Gastrulation movements seem to be impaired in such coinjected embryos. These antagonists of FGF signaling have no effect on untreated *ich *embryos (J-L and P-R). Wild-type embryos (A-C) are shown in a dorsal view, *ich *embryos (D-U) in lateral view. All embryos are at ~10 hpf. Click here for file

Additional file 6 **Figure S6 - Posterior movement of mesodermal cells only observed in 'ciuffo' embryos**. Lateral views of live, 12.5-13 hpf *ich *embryos untreated (A) or treated with bmp2bMO (B), βcat1MO (C) and bmp2bMO and βcat1MO (D). Note the clear vegetal migration of cells observable in 'ciuffo' embryos (C), and the relatively symmetric distribution of tissues between the animal and vegetal poles of bmp2bMO (co-)injected embryos (B,D). Click here for file
