# Supplementary figures and images for: Correct anteroposterior patterning of the zebrafish neurectoderm in the absence of the early dorsal organizer
Source: BMC Dev Biol. 2011 May 16;11:26. doi: 10.1186/1471-213X-11-26 (PMC3120780; doi:10.1186/1471-213X-11-26)

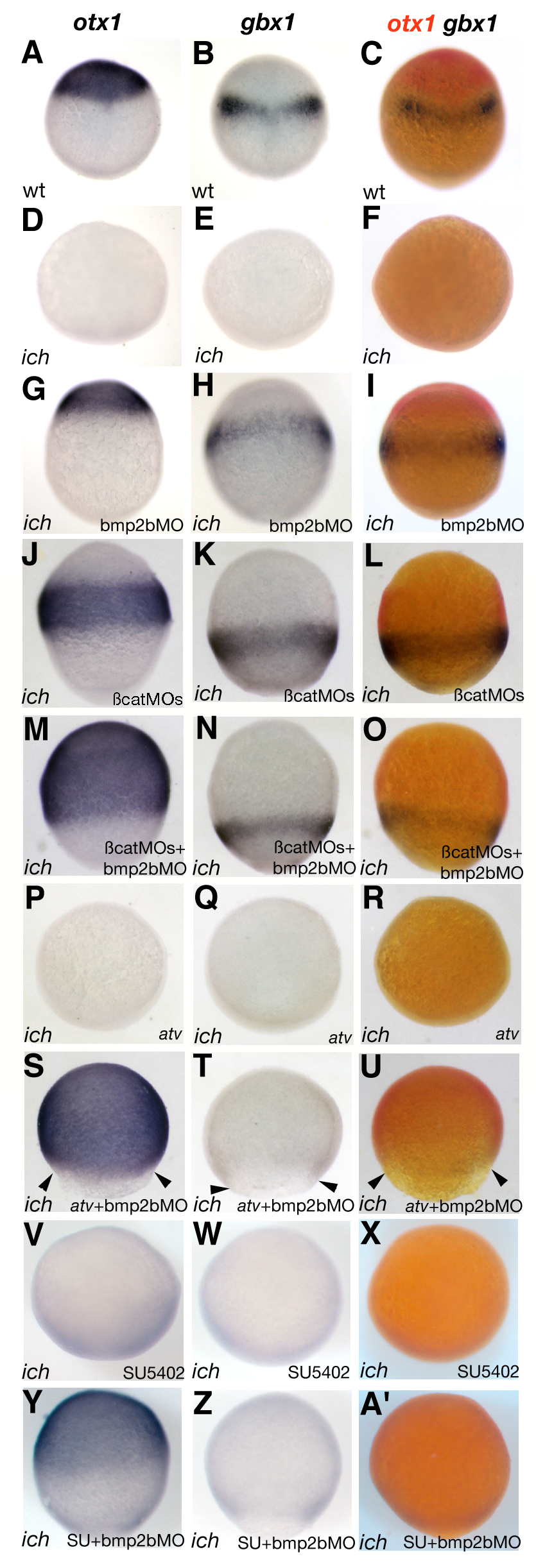

Supplement: Additional file 1 — Figure S1 - Wnt-, TGFβ- and FGF-signaling have pivotal roles in the posteriorization of the neuroectoderm of embryos both inhibited in BMP signaling and devoid of organizer activity. Single in situ hybridization with otx1 (A,D,G,J,M,P,S,V,Y) or gbx1 (B,E,H,K,Q,T,W,Z) probes or double in situ hybridization with both probes (C,F,I,L,O,R,U,X,A') is shown for wild-type embryos (A-C), untreated ich embryos (D-F), or ich embryos treated with bmp2bMO (G-I), the two β-cat MOs (J-L), bmp2bMO plus the two β-cat MOs (M-O), or ich embryos injected with antivin mRNA (P-R), antivin mRNA plus bmp2bMO (S-U), SU5402 (V-X), or SU5402 plus bmp2bMO (Y-A'). Wild-type embryos [A-C] are shown in dorsal views, while ich embryos are shown in lateral views. All embryos are at ~100% epiboly. Arrowheads in the antivin (atv) treated embryos point to the edge of the germ-ring. [file 1471-213X-11-26-S1.PNG]

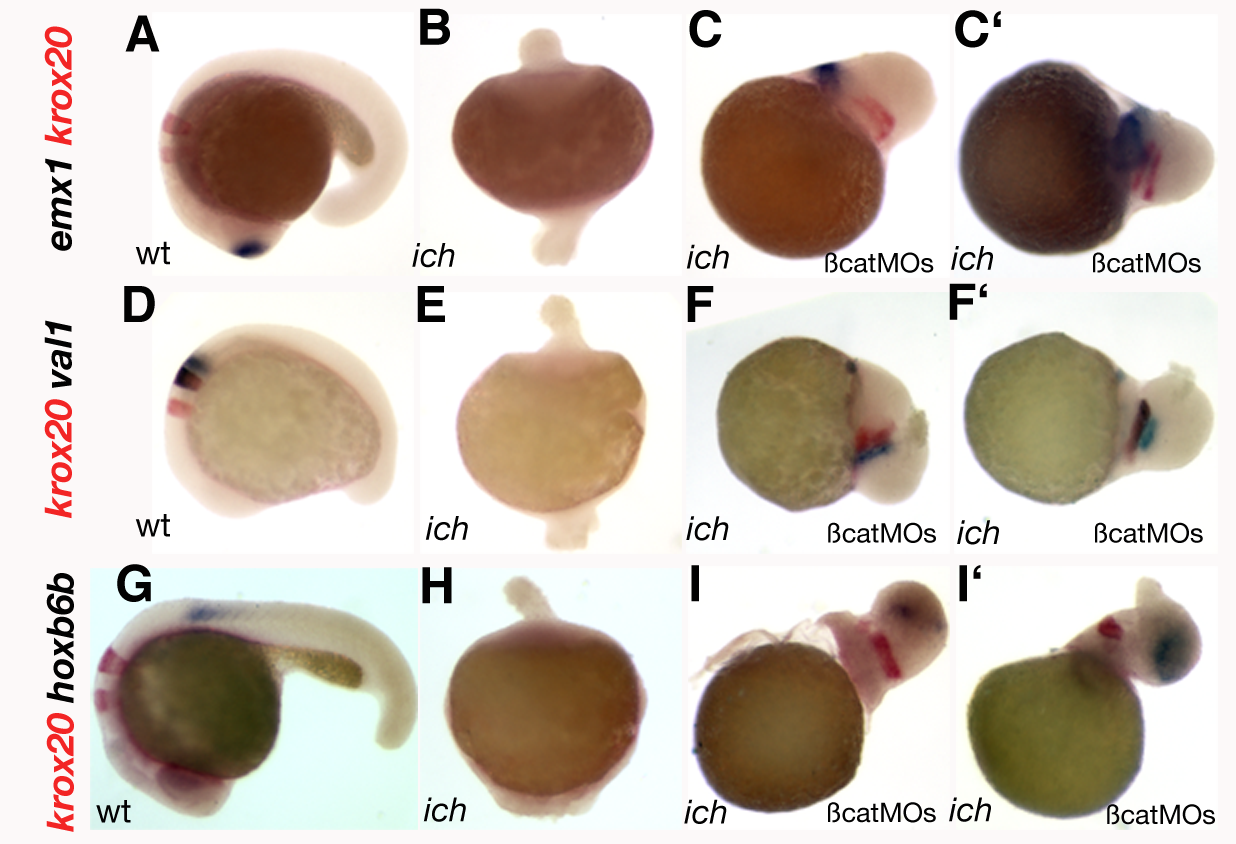

Supplement: Additional file 2 — Figure S2 - Complete repression of β-catenin signaling in ich embryos induces neuroectoderm with correct AP pattern. Untreated ich embryos do not express emx1, krox20, val, or hoxb6b (B,E,H), while their siblings coinjected with βcatMOs (C,C',F,F',I,I') express these neuroectodermal markers in a correct order at 22 hpf, with the anterior to posterior direction corresponding to proximal to distal relative to the yolk (compare A with C,C', D with F, F', and G with I,I'). The following probe-pairs were used: emx1 (blue) and krox20 (red) (A-C'), krox20 (red) and val (blue) (D-F'), and krox20 (red) and hoxb6b (blue) (G-I'). [file 1471-213X-11-26-S2.PNG]

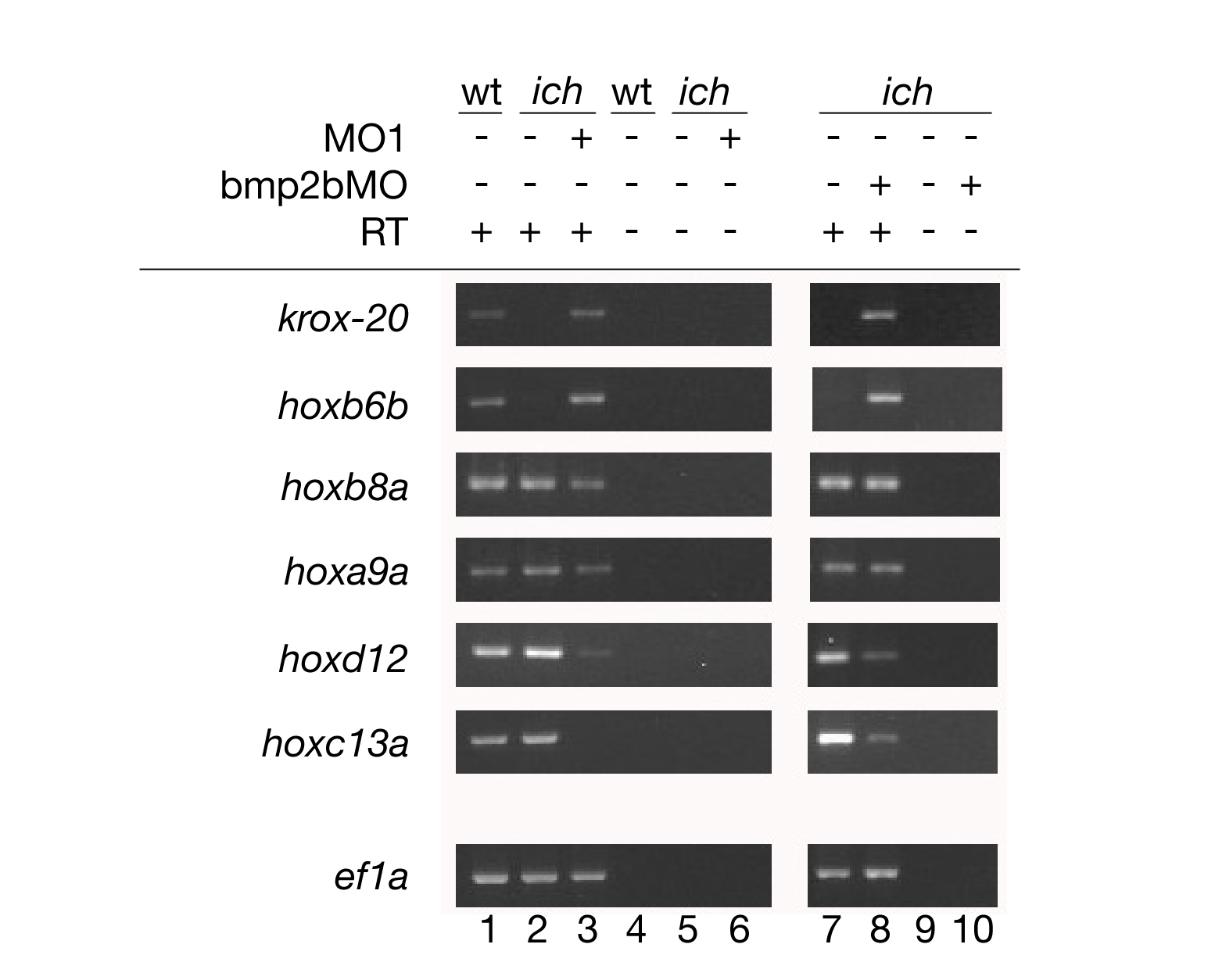

Supplement: Additional file 3 — Figure S3 - Inhibition of Wnt- or BMP signaling in ich embryos induces typical anterior neurectodermal markers. RT-PCR amplification of characteristic neurectodermal patterning markers (krox20, hoxb6b, hoxb8a, hoxa9a, hoxd12, hoxc13a) was carried out using oligo dT-primed cDNA samples from wild-type embryos (lanes 1-4), uninjected ich embryos (lanes 2,5,7,9), or ich embryos injected with βcat1MO ('ciuffo') (lanes 3,6) or bmp2bMO embryos (lanes 8,10). Whereas in ich embryos, only the posterior-most hox markers are present (lanes 2,7), 'ciuffo' embryos expressed almost the complete range of neurectodermal patterning markers examined, except that expression of hoxc13a, the most posterior marker examined, is absent, and the level of hoxd12a is reduced (lane 3). In contrast, bmp2bMO injection induced expression of all assayed neurectodermal markers (lane 8). Controls lacking RT were performed to make sure the signals observed were dependent on RNA (lanes 4-6,9,10). [file 1471-213X-11-26-S3.PNG]

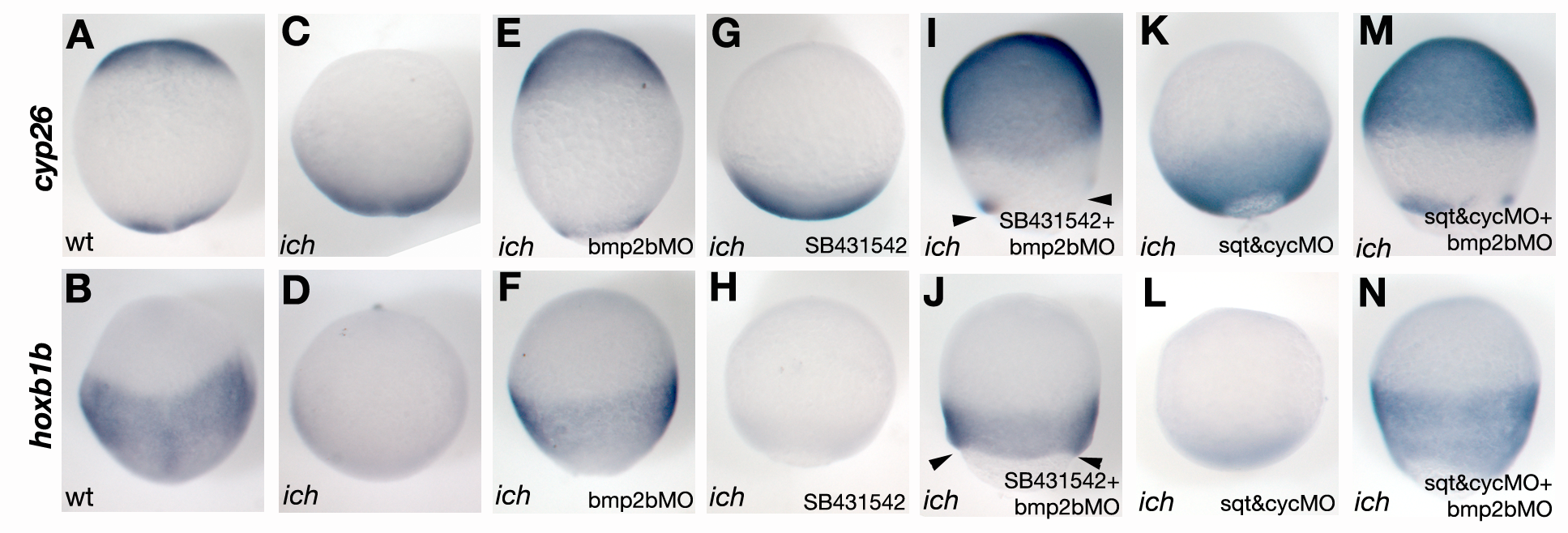

Supplement: Additional file 4 — Figure S4 - Posteriorizing TGFβ activity is only partly dependent on Nodal signals. In situ hybridization with cyp26 (A,C,E,G,I,K,M) or hoxb1b (B,D,F,H,J,L,N) probes is shown for wild-type embryos (A,B), untreated ich embryos (C,D), ich embryos treated with bmp2bMO (E,F), SB431542 (G,H), sqtMO and cycMO (K,L), or bmp2bMO in combination with SB431542 (I,J) or sqtMO and cyc MO (M,N). A small molecular inhibitor of the TGFβ pathway, SB431542, has no effect on untreated ich embryos (G,H), but when it is applied to bmp2bMO injected ich embryos, it anteriorizes the neuroectoderm (I,J). These embryos have gastrulation defects; the arrowheads point to the position of the stalled germ-ring. Coinjection of sqtMO and cycMO with bmp2bMO results in a slight expansion of the cyp26 domain (M), and a mild reduction of the hoxb1b domain (N), showing that the posteriorizing effects of TGFβ signaling are dependent to some extent on Nodal-independent signals. Injection of sqtMO along with cycMO into untreated ich embryos has no effect on cyp26 and hoxb1b expression (K,L). SB431542 was used at 2.4 mM concentration; sqtMO and cycMO were 3 mM each. Wild-type embryos (A,B) are shown in a dorsal view, ich embryos (C-N) from a lateral view. All embryos are at ~10 hpf. [file 1471-213X-11-26-S4.PNG]

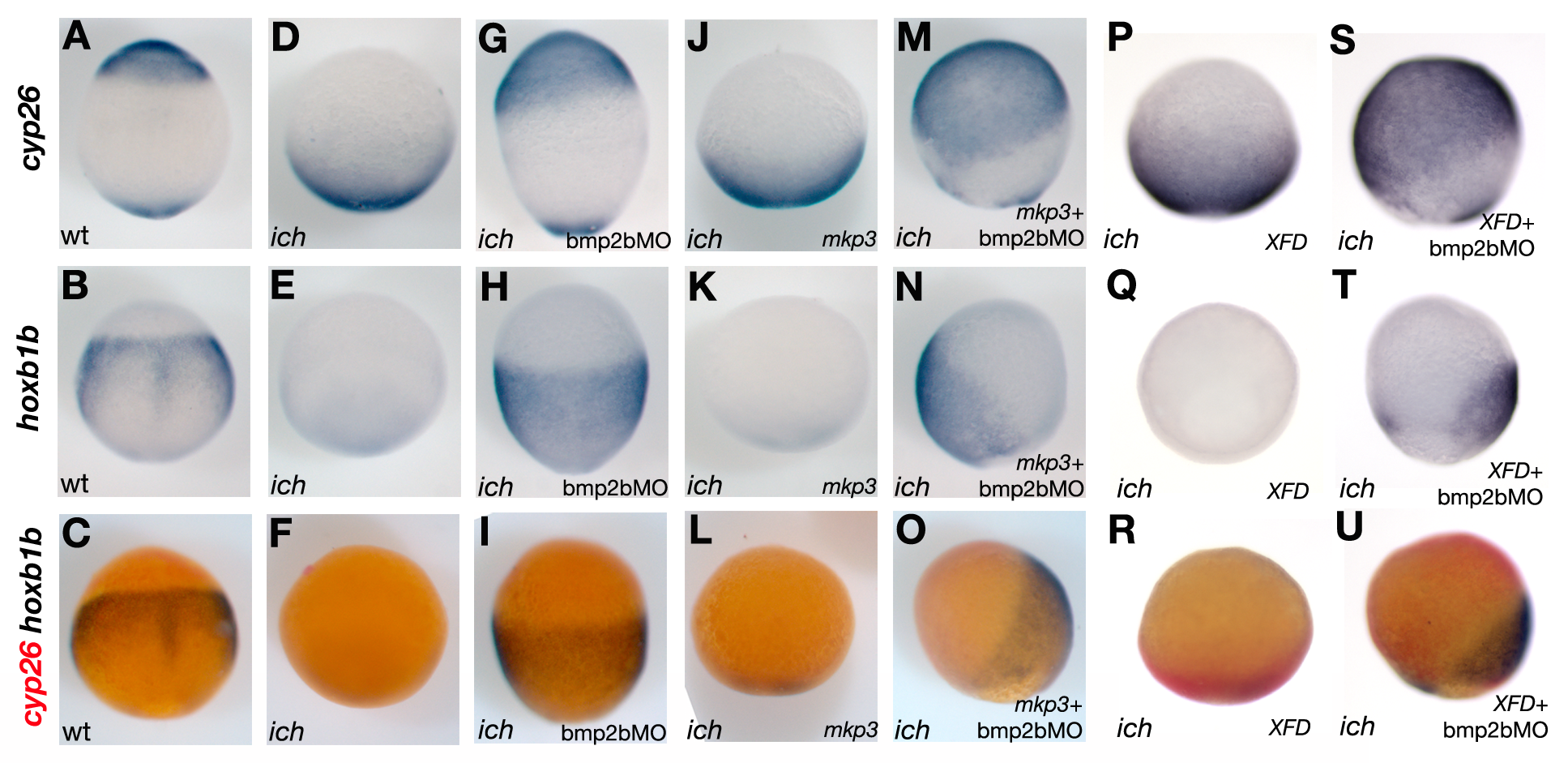

Supplement: Additional file 5 — Figure S5 - Antagonists of the FGF pathway can anteriorize ich embryos with impaired BMP signaling. Single in situ hybridization with cyp26 (A,D,G,J,M,P,S) or hoxb1b (B,E,H,K,N,Q,T) probes or double in situ hybridization with both probes (C,F,I,L,O,R,U) is shown for wild-type embryos (A-C), untreated ich embryos (D-F), ich embryos treated with bmp2bMO (G-I), injected with XFD or mkp3 mRNAs alone (J-L and P-R), or in combination with bmp2bMO (M-O and S-U). The injection of mRNAs encoding a dominant negative FGF receptor, XFD, or a negative regulator of the MAPK pathway, mkp3, into bmp2bMO-injected embryos results in the posterior expansion of the anterior neurectodermal marker, cyp26 (M,O, and S,U, and a reduction of the posterior neurectodermal domain, marked by hoxb1b (N,O and T,U). Gastrulation movements seem to be impaired in such coinjected embryos. These antagonists of FGF signaling have no effect on untreated ich embryos (J-L and P-R). Wild-type embryos (A-C) are shown in a dorsal view, ich embryos (D-U) in lateral view. All embryos are at ~10 hpf. [file 1471-213X-11-26-S5.PNG]

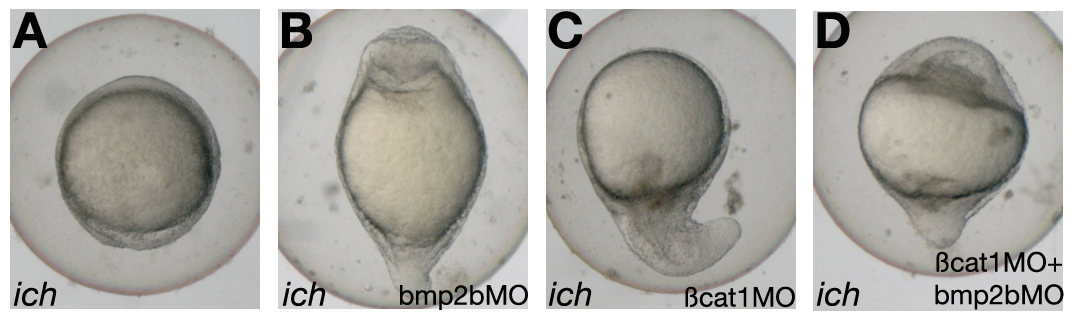

Supplement: Additional file 6 — Figure S6 - Posterior movement of mesodermal cells only observed in 'ciuffo' embryos. Lateral views of live, 12.5-13 hpf ich embryos untreated (A) or treated with bmp2bMO (B), βcat1MO (C) and bmp2bMO and βcat1MO (D). Note the clear vegetal migration of cells observable in 'ciuffo' embryos (C), and the relatively symmetric distribution of tissues between the animal and vegetal poles of bmp2bMO (co-)injected embryos (B,D). [file 1471-213X-11-26-S6.PNG]
